# Effect of sagittal alignment on patient outcomes following total knee replacement: A systematic review and correlation analysis

**DOI:** 10.1002/jeo2.70731

**Published:** 2026-05-04

**Authors:** Prakrit R. Kumar, Daya K. Sumra, Aurora Segre Carnell, Ranya V. Kumar, Umar Said, Amr Selim, Muhamed M. Farhan‐Alanie, Helen Parsons, Andrew Metcalfe

**Affiliations:** ^1^ Warwick Medical School University of Warwick Coventry UK; ^2^ Department of Trauma and Orthopaedics Surgery University Hospital Coventry & Warwickshire Coventry UK; ^3^ School of Clinical Medicine University of Cambridge Cambridge UK; ^4^ School of Medicine, Keele University Staffordshire UK

**Keywords:** patient outcomes, proms, sagittal alignment, systematic review, total knee replacement

## Abstract

**Purpose:**

With enhanced implant precision facilitated by robotic technologies, there is renewed attention on the contribution of total knee replacement (TKR) alignment to the 20% post‐operative dissatisfaction rate. Although coronal alignment has been extensively studied, the impact of post‐operative sagittal alignment on patient‐reported outcome measures (PROMs) remains unclear. This review addresses this gap.

**Methods:**

We searched five electronic databases (inception‐15 February 2025) for studies reporting post‐TKR sagittal alignment and PROMs. Case‐weighted regression models examined sagittal alignment—PROM relationships at individual post‐operative timepoints, across pooled timepoints and using time‐adjusted aggregated analyses. Risk of Bias was assessed using ROB2 and ROBINS‐I V2 tools.

**Results:**

Of 622 studies, 52 were included (*n *= 11,180 TKRs, 9431 patients). Most (92%) studies reported only one or two sagittal parameters, with fewer than five assessing more than two, and measurement protocols varied widely. Higher posterior‐tibial‐slope (PTS) was associated with improved visual analogue scale pain scores at 1 month (regression‐coefficient [RC] = 1.00, *p* < 0.001, *n* = 90 TKR, PTS range: 6.80–6.90), Oxford‐knee‐score (OKS) at 6 months (RC = 3.28, *p* = 0.009, *n* = 117, PTS range: –5.00 to 4.38) and pooled OKS across all timepoints. Higher femoral flexion (FF) was associated with improved OKS at 24 months (RC = 4.05, *p* = 0.004, *n* = 539, FF range: 3.00–4.96) and improved knee‐society‐score‐overall (KSS‐overall), KSS‐function, short‐form‐survey‐12 and knee‐injury‐and‐osteoarthritis‐outcome‐score. Lower femoral‐sagittal‐angle (FSA; range: 1.30–3.80) was significantly associated with improved KSS‐knee at 12 months (RC = –6.20, *p* = 0.008, *n* = 160), and when pooled across timepoints (RC = –5.32, *p* < 0.001, *n* = 280), and in time‐adjusted aggregated analysis (RC = –6.09, *p* < 0.001, *n* = 280). Higher posterior‐condylar‐offset (PCO) was associated with improved KSS‐overall at 12 months (RC = 42.945, *p* = 0.002, *n* = 613, PCO range: 30.40–33.60 mm), higher KSS‐knee (RC = 0.67, *p* = 0.019, *n* = 175, PCO range: 24.5–33.6 mm) in pooled analysis, and improved KSS‐overall (RC = 31.64, *p* < 0.001, *n* = 2338, PCO range: 24.0–33.6 mm) in time‐adjusted analysis.

**Conclusion:**

This review provides the first quantitative synthesis linking post‐operative sagittal alignment parameters with PROMs. Higher posterior‐tibial‐slope, femoral flexion, and posterior‐condylar offset, and lower femoral‐sagittal angle were each significantly associated with improved PROMs, underscoring the importance of sagittal alignment beyond the coronal plane. However, heterogeneity in measurement protocols, underreporting of parameters limit comparability and generalisability. Standardised, multiplanar reporting is essential to help define evidence‐based alignment associations and inform surgeons about how best to improve patient outcomes.

**Level of Evidence:**

Level I.

**Abbreviations**
ACO: anterior condylar offset
DFSAA: distal femoral sagittal anteverted angle
EQ‐5D: EuroQol‐5D
FEA: flexion/extension angle
FFA: femoral flexion angle
FJS: forgotten joint score
FSA: femoral sagittal angle
HSS: hospital for special surgery knee‐rating scale
KOOS: knee injury and osteoarthritis outcome score
KSS: knee society knee society score
OA: osteoarthritis
OKS: Oxford knee score
PCO: posterior condylar offset
PRISMA: Preferred Reporting Items for Systematic Reviews and Meta‐Analysis
PROMs: patient reported outcome measures
PROSPERO: prospective register of systematic reviews
PTS: posterior tibia slope
ROB2: risk of bias version 2
ROBINS‐I V2: risk of bias in non‐randomised studies of interventions version 2
SF‐12: short‐form‐12‐health‐survey
SF‐36: short‐form‐36‐health‐survey
TKR: total knee replacement
WOMAC: Western Ontario and McMaster Universities Osteoarthritis Index

## INTRODUCTION

Knee osteoarthritis (OA) accounts for nearly 80% of the global OA burden and is a leading cause of pain, disability and reduced quality of life [10]. Total knee replacement (TKR), which involves replacing the damaged joint surfaces with implants, is the most cost‐effective definitive treatment for knee OA [2, 5, 6, 28, 46, 49, 58]. However, up to 20% of people are dissatisfied after a TKR, with 25% of patients regretting their decision to have undergone the surgery and 55% reporting residual symptoms at 1 year post‐operatively [7, 17, 20, 29, 35, 43]. These are high dissatisfaction rates compared to other joint‐replacement operations, such as total hip replacement [15, 35]. One potential reason for these issues is implant position (alignment). This can influence the extent and degree of stretching of the soft tissues, knee range of motion and gait [2, 12, 26, 52]. Thus, it is believed that optimising limb alignment can improve patient outcomes [12, 34, 41, 42]. Importantly, recent advances in robotic technology enable surgeons to achieve greater precision in intended limb alignment and implant positioning, meaning these are modifiable surgical factors [11, 22, 25, 27, 48]. However, although robotics enables surgeons to reliably achieve a planned target alignment, the optimal alignment remains uncertain, raising the risk of being ‘precisely wrong’.

Whilst the impact of post‐operative coronal alignment on patient‐reported outcome measures (PROMs) has been extensively studied, likely due to its ease of visualisation and measurement [13, 24, 33], sagittal alignment has received comparatively less attention. This is despite being recognised as a critical determinant of knee biomechanics and patient function after TKR [19]. Excessive deviations in sagittal plane can disrupt knee kinematics, increase ligament strain, and reduce the range of motion needed for daily activities [4]. On the tibial side, excessive posterior slope may limit full knee extension, while an anterior tibial slope can restrict flexion, both leading to abnormal gait and stance‐phase instability [30]. On the femoral side, a hyperextended femoral component increases patellofemoral joint stress, risks anterior cortical notching, and thereby possibly contributes to patellofemoral dysfunction and chronic anterior knee pain [37, 44]. Conversely, excessive femoral component flexion can disrupt gap balance, resulting in uneven ligament tension and abnormal joint movements [37]. Despite its biomechanical and functional importance [23] the impact of sagittal alignment on patient‐reported outcomes remains underexplored.

To address this literature gap, we completed a review that aimed to: (A) explore how sagittal alignment parameters following TKR are reported including which parameters are measured, the methods and protocols used, and (B) systematically review and quantitatively assess the relationship between different post‐TKR sagittal alignment and PROMs [45].

## METHODS

### Protocol registration

The study protocol was registered with the Prospective Register of Systematic Reviews (PROSPERO). The final review methodology adhered to the registered protocol without deviation. This review addresses sagittal alignment, while coronal and axial planes are reported in separate companion papers, with searches conducted independently for each. This study adhered to the Preferred Reporting Items for Systematic Reviews and Meta‐Analysis (PRISMA) 2020 guidelines (Appendices S1 and S2) [40].

### Search methodology

Medline, Embase, Scopus, Web of Science and the Cochrane Database of Systematic Reviews were searched from inception to 15 February 2025. The complete search and Medical Subject Heading and Boolean operator terms are shown in Appendix S3.

### Eligibility criteria

Studies were included if they met the following criteria: (1) randomised controlled trials (RCTs), case–control studies, and prospective or retrospective cohort studies investigating either unilateral or bilateral primary total knee arthroplasty performed conventionally or with the assistance of robotic‐assisted techniques or manual techniques (2) primary indication for TKR was primary OA, (3) English full‐text manuscript with available data, (4) reported one or more of the following sagittal alignment parameters: Posterior tibia slope (PTS), femoral flexion angle (FFA), femoral sagittal angle (FSA), posterior condylar offset (PCO), anterior condylar offset (ACO), distal femoral sagittal anteverted angle (DFSAA), flexion/extension angle (FEA); (5) reported one of the following validated PROMs (including but not limited to knee society knee society score [KSS], forgotten joint score [FJS], Oxford knee score [OKS] and Western Ontario and McMaster Universities Osteoarthritis Index [WOMAC], hospital for special surgery knee‐rating scale [HSS], knee injury and osteoarthritis outcome score [KOOS], short‐form‐12‐health‐survey [SF‐12], short‐form‐36‐health‐survey [SF‐36], EuroQol‐5D [EQ‐5D]). Studies were excluded if they were case reports, conference abstracts, non‐human studies, reviews, editorials, or did not report relevant sagittal alignment parameters or validated PROMs.

### Data extraction

Two reviewers independently screened articles based on title and abstract using Rayyan, followed by full‐text review of selected articles [39]. Data from included studies were then extracted into a pre‐templated form by the two reviewers.

### Risk of bias assessment

Risk of bias was assessed by two authors using the cochrane risk‐of‐bias version 2 (ROB2) tool for RCTs [51] and the risk‐of‐bias‐in‐non‐randomised‐studies‐of‐interventions version 2 (ROBINS‐I V2) for non‐randomised studies [50].

### Statistical analysis

For each study, mean values for sagittal alignment parameters, PROMs, numbers of TKRs reported, and corresponding follow‐up timepoints were extracted. Where data were reported as medians and interquartile ranges, this missing data were estimated and converted to means and standard deviations using the DECOMA cochrane tool [8], study‐level descriptive statistics were then constructed to summarise the number of studies, and the mean and range of sagittal parameters and PROMs at specific timepoints.

Following Valkering et al.'s methodology [53], study‐level linear regression analyses were conducted to assess the relationship between mean sagittal alignment parameters and mean PROMs at each timepoint. All analysis in this paper were weighted by number of TKA. Subsequently, weighted pooled analyses combined mean sagittal parameters and PROMs across all timepoints to evaluate overall associations. Finally, to adjust for temporal effects, time‐adjusted aggregated weighted regression models were fitted incorporating PROM time from surgery as a covariate. In summary, analyses were conducted at individual timepoints, pooled across timepoints and adjusted for time. Multivariable linear regression was not feasible as none of identified studies reported all sagittal alignment parameters comprehensively. Regression coefficients, 95% confidence intervals and *p*‐values were calculated, with statistical significance defined as *p* < 0.05. All analyses were performed using R (version 2024.12.1+563).

## RESULTS

### Study characteristics

**Figure 1:**
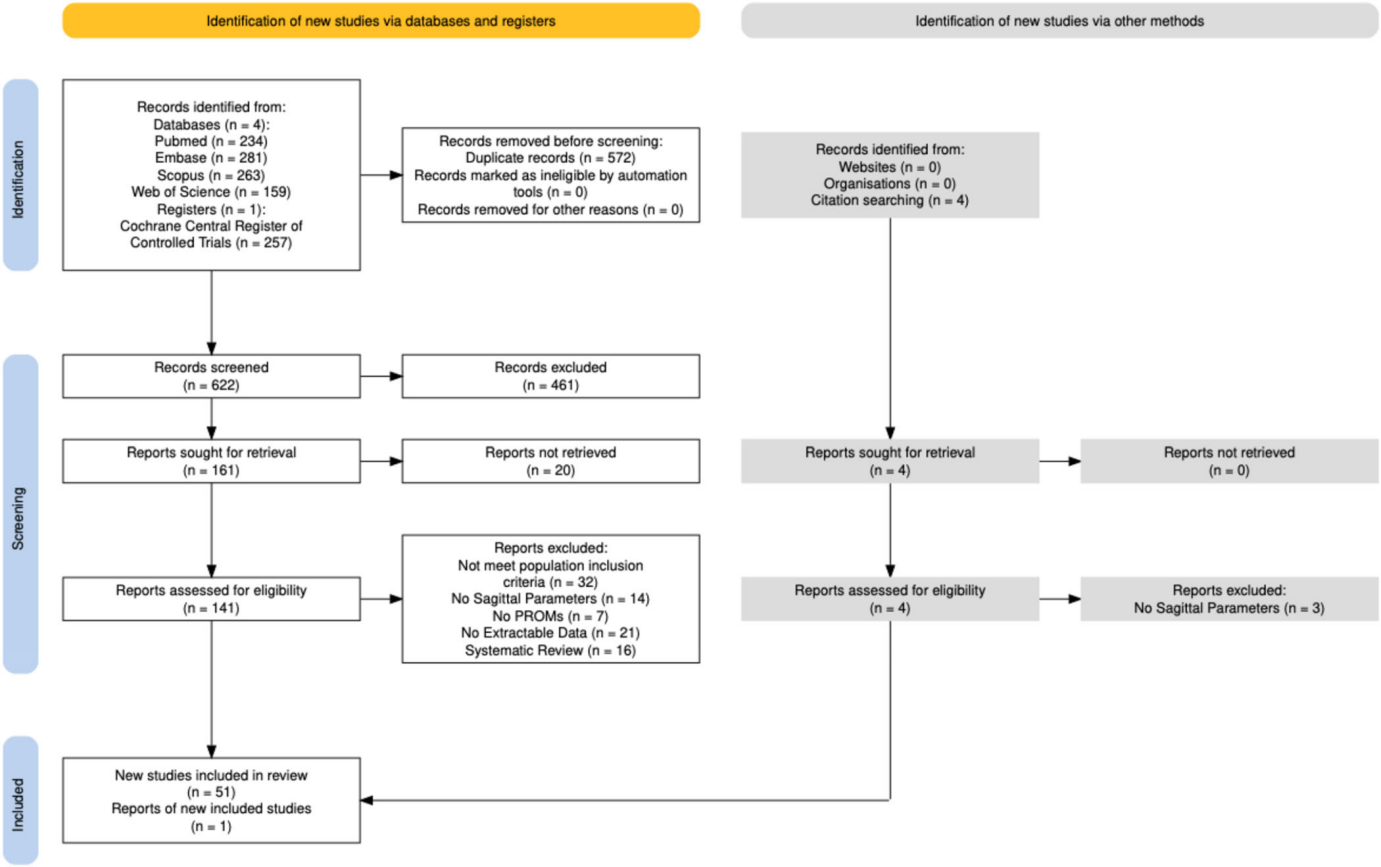
Preferred Reporting Items for Systematic Reviews and Meta‐Analysis flowchart.

The search identified 1194 potentially eligible articles; after removing duplicates, 622 remained for screening. Title and abstract screening narrowed these to 141 for full‐text review. Fifty‐one studies met the inclusion criteria, and one additional study was identified through comprehensive examination of full texts of eligible studies, resulting in 52 included studies (Figure 1) [21]. Our review included 18 RCTs and 34 cohort studies (Table 1), comprising of 11,180 TKRs and 9431 patients (mean age: 68.0 years, mean BMI: 29.3 kg/m^2^, among 49 and 41 studies reporting these data).

**Table 1:** Characteristics of included studies.

ID	Study	Year	Country of study	Study design	Retrospective versus prospective	Level of evidence	Number of patients (*n* = 9307)	Number of TKA (*n* = 11,026)	Measurement method
2	Eggermont et al.	2025	Belgium	Cohort	Prospective	3	266	192	XR
3	Nedopil et al.	2025	USA	Cohort	Retrospective	3	99	562	CT
4	Bhattacharjee et al.	2024	India	Cohort	Prospective	2	27	27	XR
7	Wang et al.	2024	China	RCT	Prospective	1	113	120	CT
8	Cacciola et al.	2024	Italy	Cohort	Retrospective	3	95	95	XR
12	Van de Kelft et al.	2023	Belgium	Cohort	Retrospective	3	91	98	XR
13	Ersin et al.	2023	Turkey	Retrospective	Cohort	3	124	154	XR
14	Albelooshi et al.	2023	UAE	Cohort	Retrospective	3	151	302	XR
17	Hao et al.	2023	China	Cohort	Retrospective	3	90	90	XR
23	Okayoshi et al.	2023	Japan	Cohort	Retrospective	3	Not reported	60	XR
26	Shah et al.	2022	USA	Cohort	Prospective	2	27	27	XR
27	Mahoney et al.	2022	USA	Cohort	Prospective	2	0	229	Intra‐operative
28	Shatrov et al.	2022	Australia	Cohort	Retrospective	3	476	476	XR
30	Zheng et al.	2022	China	Cohort	Retrospective	3	389	389	XR
33	Narkbunnam et al.	2022	Thailand	RCT	Prospective	1	60	60	CT
34	Gao et al.	2021	China	Cohort	Retrospective	3	98	102	XR
35	Scott et al.	2021	Scotland	Cohort	Prospective	2	Not reported	539	XR
41	Rassir et al.	2019	Netherlands	Cohort	Retrospective	3	304	304	XR
48	Lee	2019	South Korea	Cohort	Retrospective	3	65	94	XR
50	Behrend et al.	2019	Switzerland	Cohort	Retrospective	3	260	282	XR
52	Clement et al.	2018	Scotland	Cohort	Retrospective	3	295	295	XR
54	Kosse et al.	2018	Netherlands	RCT	Prospective	1	42	42	XR
57	Wang et al.	2015	China	Cohort	Prospective	2	89	89	XR
69	Cho et al.	2025	South Korea	Cohort	Prospective	2	100	100	XR
72	Xing et al.	2024	China	Cohort	Retrospective	3	120	120	XR
77	Singh et al.	2024	India	Cohort	Prospective	2	152	152	XR
81	Aydin et al.	2023	Turkey	Cohort	Retrospective	3	100	110	XR
82	Luan et al.	2022	China	Cohort	Retrospective	3	50	50	NA
83	Dhaliwal et al.	2022	USA	Cohort	Prospective	2	42	42	CT
84	Bar Ziv et al.	2022	Israel	Cohort	Retrospective	3	674	674	XR
85	Jagadeesh et al.	2022	India	RCT	Prospective	1	70	70	XR
87	Williams et al.	2021	Canada	Cohort	Retrospective	3	498	498	XR
89	Chang et al.	2020	Korea	Cohort	Retrospective	3	184	184	XR
90	Watanabe et al.	2022	Japan	Cohort	Prospective	2	44	44	XR
91	Panciera et al.	2022	Italy	Cohort	Retrospective	3	121	121	XR
93	Lee et al.	2023	South Korea	Cohort	Retrospective	3	164	164	NA
94	Yildiz et al.	2022	Turkey	RCT	Prospective	1	272	272	XR
95	Hinarejos et al.	2022	Spain	RCT	Prospective	1	115	115	XR
96	Shi et al.	2021	China	Cohort	Retrospective	3	233	233	XR
98	Kang et al.	2021	South Korea	Cohort	Retrospective	3	551	551	XR
102	Cip et al.	2018	Austria	RCT	Prospective	1	69	69	NA
107	Cip et al.	2014	Austria	RCT	Prospective	1	400	400	XR
110	Verburg et al.	2016	Netherlands	RCT	Prospective	1	100	100	NA
111	Leeuwen et al.	2017	Norway	RCT	Prospective	1	94	94	CT
112	Kim et al.	2020	South Korea	RCT	Prospective	1	1348	1448	CT
113	Dossett et al.	2012	USA	RCT	Prospective	1	82	82	CT
118	Tian et al.	2023	China	RCT	Prospective	1	123	123	CT
125	Mehdipour et al.	2020	Iran	RCT	Prospective	1	12	12	XR
131	Ensini et al.	2007	Italy	RCT	Prospective	1	214	240	NA
133	Kim et al.	2010	Korea	RCT	Prospective	1	146	292	XR
138	Öztürk et al.	2023	Turkey	RCT	Prospective	1	93	93	XR
163	Young et al.	2016	New Zealand	RCT	Prospective	1	99	99	CT

Abbreviations: CT, computer tomography; NA, not applicable; RCT, randomised controlled trial; XR, X‐Ray.

### Risk of bias assessment

Of the 18 RCTs, 14 RCTs were rated as ‘low’ risk of bias, two as having ‘some concerns’, and two with insufficient information (Appendix S4). Of the 34 cohort studies, 13 studies were rated as having an overall ‘low’ or ‘moderate’ risk of bias, while 21 were rated as ‘serious’ or ‘critical’ (Appendix S5).

### Aim A: Reporting standards

Of the six main sagittal alignment parameters described in the methods section, most studies reported only one (*N* = 27; 52%) or two (*N* = 21; 40%) parameters, with two studies reporting three and two reporting four parameters. The most frequent sagittal alignment parameters reported in the literature were posterior tibial slope (PTS, *N* = 38) and posterior condylar offset (PCO, *N* = 10) (Table 2). Different measurement protocols and definitions were described across studies, particularly for FF and FSA (details given in Appendix S6). For example, FF was measured against three different reference points: the anterior flange of the femoral prosthesis (FF1, *N* = 9 studies), the bottom of the femoral component (FF2, *N* = 4), or a line perpendicular to the femoral prosthesis (FF3, *N* = 2) [14, 36, 55]. Sagittal parameters were predominantly measured using radiographs (*N* = 37 studies), with nine studies utilising CT imaging (Table 1).

**Table 2:** Summary of sagittal alignment parameters.

Sagittal parameter	Mean (TKA‐weighted)	Minimum	Maximum	Number of studies	Number of TKAs
Posterior tibia slope	3.31	−5.00	9.19	39	8180
Distal femoral sagittal anteverted angle	4.15	4.10	4.20	1	120
Femoral flexion protocol 1	3.43	1.50	9.80	9	2031
Femoral flexion protocol 2	88.8	86.0	90.7	4	572
Femoral flexion protocol 3	2.02	0.40	3.20	2	384
Femoral sagittal angle 1	1.53	0.90	3.80	4	494
Femoral sagittal angle 2	2.05	1.21	2.31	1	102
Femoral sagittal angle 3	−5.86	−6.50	−5.30	1	94
Flexion extension angle	−1.70	−1.70	−1.70	1	50
Posterior condylar offset	26.0	24.0	33.6	10	3383
Posterior condylar offset ratio	0.62	0.44	0.50	7	1186
Anterior condylar offset	10.3	4.19	29.5	4	1279

Abbreviation: TKA, total knee arthroplasty.

Across the 52 studies, 17 different PROMs were reported, with follow‐up ranging from 1 to 156 months post‐operatively. Most studies reported one or two PROMs (*N* = 31), with *N* = 20 reporting on three (*N* = 10) and *N* = 11 four to six PROMs. These PROMs were grouped into six joint‐specific families, each encompassing relevant subscales (e.g., the KSS family includes KSS‐knee and KSS‐function). As shown in Table 3, the KSS was the most frequently reported PROM, while a smaller number of studies also reported broader measures of health and quality of life, including VAS pain, EQ‐5D and SF‐12. Where studies reported KOOS, a single value was extracted as reported, and none specified whether it represented KOOS4.

**Table 3:** Summary of patient reported outcome measures (PROMs).

PROM	Number of studies
**Joint‐specific**	
Knee society score (KSS)	
KSS—Overall	15
KSS—Function	22
KSS—Knee	13
KSS—Symptoms	2
KSS—Satisfaction	2
KSS—Expectation	2
Western Ontario and McMaster Universities Osteoarthritis Index (WOMAC)	
WOMAC—Total	19
WOMAC—Function	2
WOMAC—Pain	2
WOMAC—Stiffness	2
Oxford knee score (OKS)	15
Knee injury and osteoarthritis outcome score (KOOS)	9
Forgotten joint score 12 (FJS‐12)	7
Hospital for special surgery (HSS)	4
Health quality	
Visual analogue scale—pain (VAS—pain)	5
EuroQol – 5D (EQ‐5D)	4
Short form survey 12	2

### Aim B: Relationship between sagittal parameters and PROMs

#### PTS

In 39 studies (*n* = 8180 TKRs) reporting PTS, higher PTS was associated with improved short‐term PROMs, including visual‐analogue‐scale‐pain scores at 1 month (RC = 1.00, *p *< 0.001, *n* = 90, PTS range: 6.80–6.90) and Oxford‐knee‐score at 6 months (RC = 3.28, *p* = 0.009, *n* = 117, PTS range: –5.00 to 4.38) (Table 4); however, no significant associations were observed at medium‐ or long‐term follow‐up (up to 60 months).

**Table 4:** Significant sagittal alignment—Patient reported outcome measures (PROMs) associations at specific timepoints.

Time (months)	Parameter	Parameter minimum	Parameter maximum	PROM	PROM minimum	PROM maximum	Regression coefficient	95% lower limit	95% upper limit	*p*‐value^a^	Number of TKA	Number of studies
1	Posterior tibia slope	6.80	6.90	VAS‐pain	4.00	4.10	1.00	1.00	1.00	<0.001	90	1
6	Posterior tibia slope	−5.00	4.38	OKS	8.00	38.9	3.28	2.67	3.89	0.009	117	2
12	Femoral flexion protocol 2	86.0	89.0	KSS‐function	75.0	81.2	1.92	1.02	2.83	0.007	190	2
12	Femoral sagittal angle 1	1.30	3.80	KSS‐knee	82.3	97.4	−6.29	−8.68	−3.90	0.008	160	2
12	Posterior condylar offset	30.4	33.6	KSS‐overall	88.9	178	31.9	19.8	43.9	0.002	613	2
24	Femoral flexion protocol 1	3.00	4.96	OKS	36.2	44.1	4.05	1.88	6.21	0.004	539	1

Abbreviations: KSS, knee society score; TKA, total knee arthroplasty; OKS, Oxford knee score; VAS, visual analogue scale.

^a^ *p*‐Value refers to the significance of the regression coefficient for association between the sagittal alignment parameter and the PROM.

**Figure 2:**
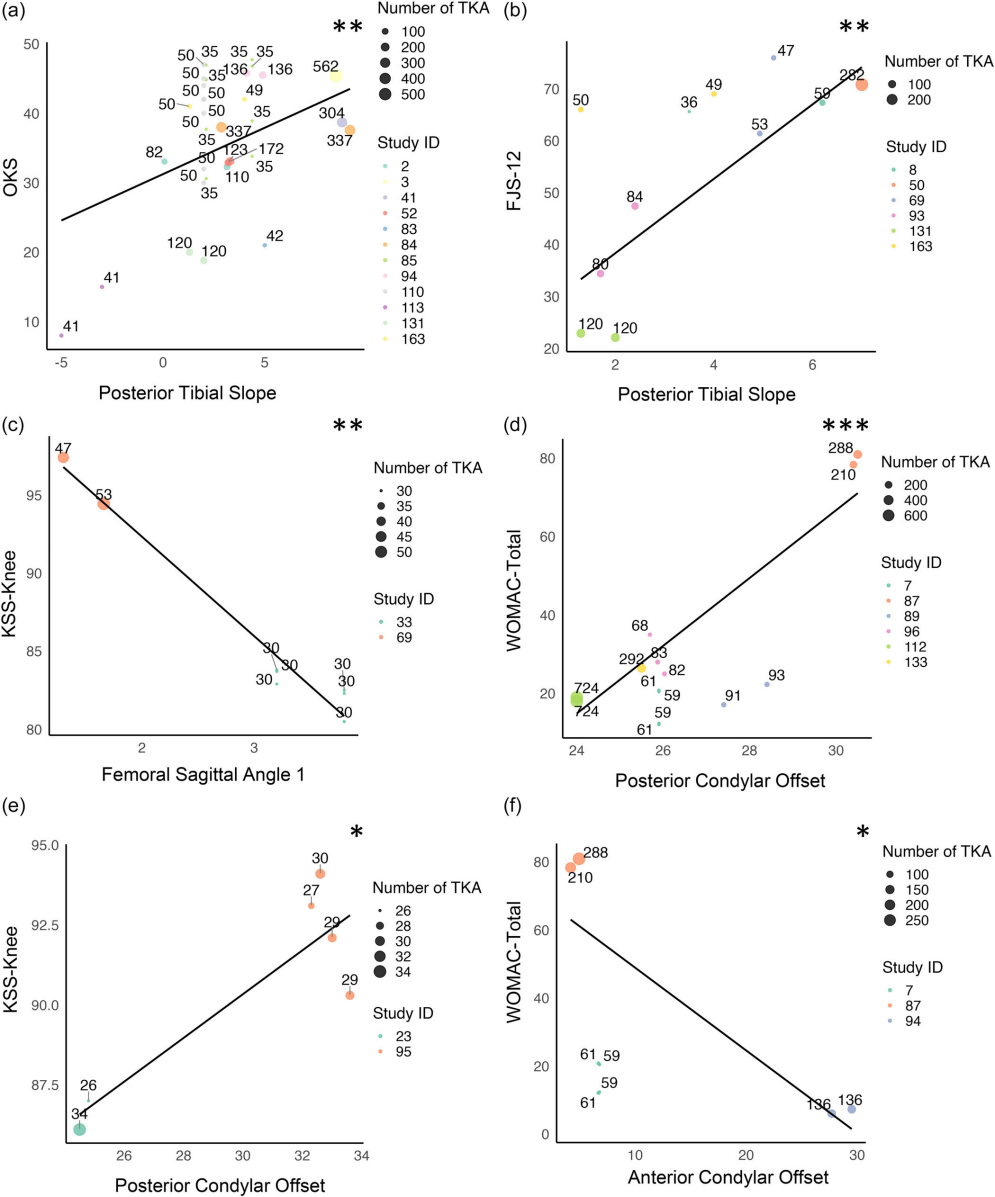
Scatterplots of significant pooled associations between sagittal alignment parameters and patient reported outcome measures (PROMs) across all timepoints. Each dot represents a study, with study ID indicated by colour. The dot size reflects number of total knee arthroplasty (TKAs), with the exact number shown above each dot. Significance: *p < 0.05; **p < 0.01; ***p < 0.001.

When pooled across all timepoints, higher PTS was associated with increased OKS (RC = 1.34, *p* = 0.002, *n* = 3442, PTS range: –5.00 to 9.90) and FJS‐12 (RC = 7.17, *p* = 0.002, *n* = 980, PTS range: 1.30–7.00) (Figure 2a,b). However, within a limited range of PTS values (3.60°–3.80°), lower PTS was associated with improved EQ‐5D scores (RC = –10.33, *p* = 0.042, *n* = 180) (Table 5). Interestingly, in the time‐adjusted aggregated analysis there was no statistically significant relationship with any of the PROMs.

**Table 5:** Significant sagittal alignment—Patient reported outcome measures (PROMs) associations pooled across timepoints.

Parameter	Parameter minimum	Parameter maximum	PROM	PROM minimum	PROM maximum	Regression coefficient	95% lower limit	95% upper limit	*p*‐value^a^	Number of TKA	Number of studies
Posterior tibia slope	−5.000	9.190	OKS	8.000	47.73	1.34	0.53	2.14	0.002	3442	12
Posterior tibia slope	1.300	7.000	FJS	22.10	75.94	7.17	3.32	11.0	0.002	980	6
Posterior tibia slope	3.600	3.800	EQ‐5D	78.80	82.80	−10.3	−20.0	‐0.63	0.042	180	1
Femoral sagittal angle 1	1.300	3.800	KSS‐knee	80.50	97.41	−6.38	−7.44	−5.32	<0.001	280	2
Femoral sagittal angle 1	3.200	3.800	EQ‐5D	78.80	82.80	−3.44	−6.68	−0.21	0.042	180	1
Posterior condylar offset	24.00	30.50	WOMAC‐total	12.10	80.90	8.64	6.04	11.2	<0.001	2895	6
Posterior condylar offset	24.50	33.60	KSS‐knee	86.10	94.10	0.69	0.19	1.18	0.019	175	2
Posterior condylar offset ratio	0.460	1.490	WOMAC‐total	12.20	83.00	62.2	52.5	71.9	<0.001	617	3
Anterior condylar offset	4.190	29.50	WOMAC‐total	6.000	80.90	−2.43	−4.70	−0.16	0.039	1010	3

Abbreviations: EQ‐5D, EuroQol‐5D; FJS, forgotten joint score; KSS, knee society score; OKS, Oxford knee score; TKA, total knee arthroplasty; WOMAC, Western Ontario and McMaster Universities Osteoarthritis Index.

^a^ *p*‐Value refers to the significance of the regression coefficient for association between the sagittal alignment parameter and the PROM.

#### Femoral flexion (FF)

FF Protocol 1 and FF Protocol 2 were reported in nine studies (*n* = 2031 TKRs) and four studies (*n* = 572 TKRs), respectively, with PROMs assessed from 1 to 24 months. In the medium term, lower FF2 was associated with better VAS‐pain at 12 months (RC = 1.92, *p* = 0.007, *n* = 190, FF2 range: 86.0–89.0), while higher FF1 was linked to greater OKS at 24 months (RC = 4.05, *p* = 0.004, *n* = 539, FF1 range: 3.00–4.96).

**Figure 3:**
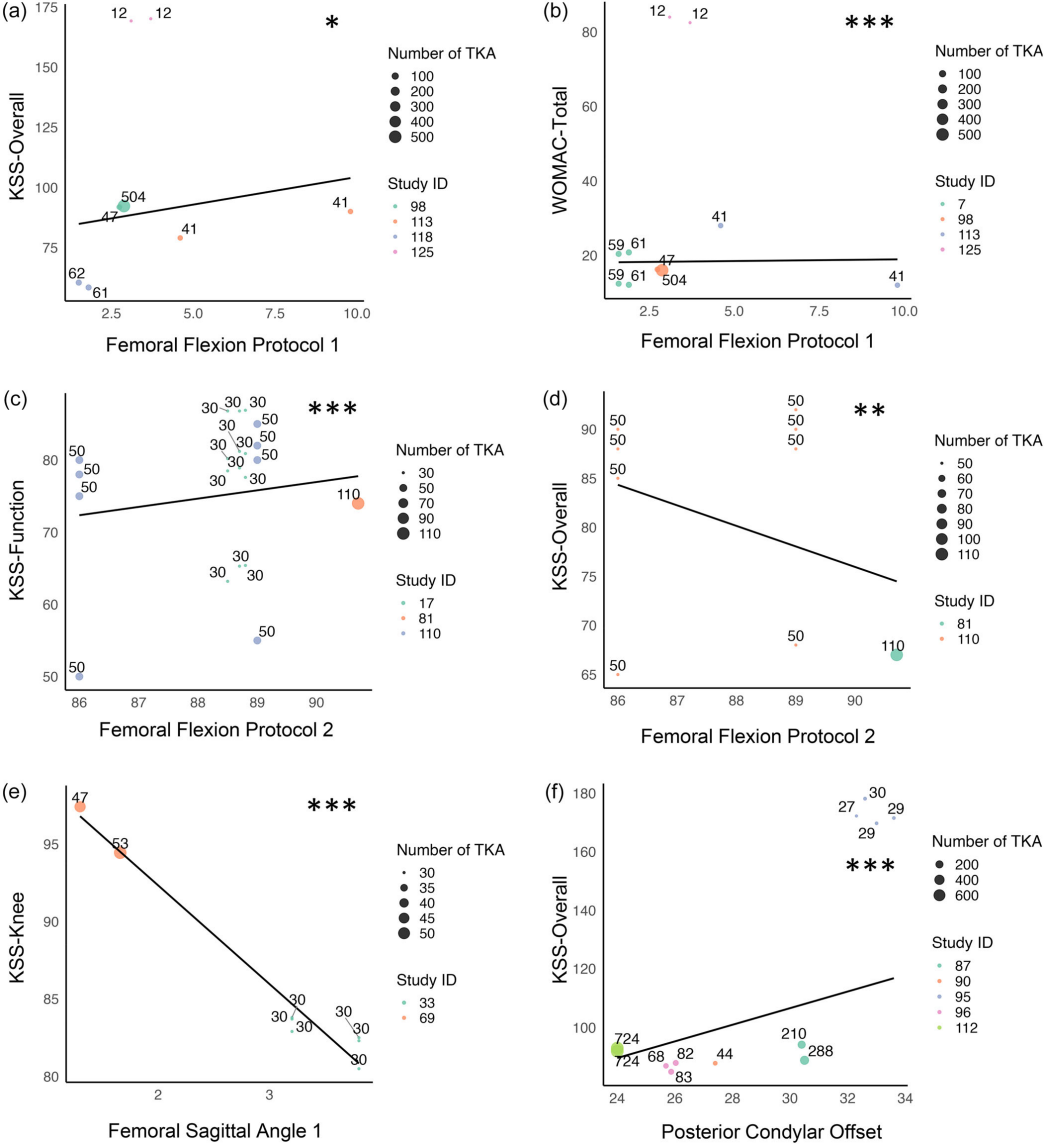
Scatterplots of significant time‐adjusted associations between sagittal alignment parameters and patient reported outcome measures (PROMs). Each dot represents a study, with study ID indicated by colour. The dot size reflects number of total knee arthroplasty (TKAs), with the exact number shown above each dot. Significance: *p < 0.05; **p < 0.01; ***p < 0.001.

Although there was no statistically significant associations in pooled analyses, time‐adjusted aggregated analysis showed higher FF1 was significantly associated with improved WOMAC‐total (RC = –3.52, *p* < 0.001, *n* = 897, FF1 range: 1.60–9.80) and KSS‐Overall (RC = 2.07, *p* = 0.011, *n* = 780, FF1 range: 1.50–9.80) (Figure 3a,b). Similarly, higher FF2 was associated with increased KSS‐Overall (RC = 0.83, *p* = 0.003, *n* = 510), KSS‐Function (RC = 1.93, *p* < 0.001, *n* = 870), SF‐12 (RC = 0.67, *p* < 0.001, *n* = 400), and KOOS (RC = 1.42, *p* = 0.011, *n* = 400), all within similar FF2 ranges (86.0–89.0) (Table 6, Figure 3c,d).

**Table 6:** Significant time‐adjusted aggregated sagittal alignment—Patient reported outcome measures (PROMs) associations.

Parameter	Parameter minimum	Parameter maximum	PROM	PROM minimum	PROM maximum	Regression coefficient	95% lower limit	95% upper limit	*p*‐value^a^	Number of TKA	Number of studies
Femoral flexion protocol 1	1.60	9.80	WOMAC‐total	12.0	84.0	−3.04	−3.52	−2.57	<0.001	897	4
Femoral flexion protocol 1	1.50	9.80	KSS‐overall	58.4	170	2.07	0.92	3.22	0.011	780	4
Femoral flexion protocol 2	86.0	90.7	KSS‐overall	65.0	92.0	0.83	0.53	1.14	0.003	510	2
Femoral flexion protocol 2	86.0	90.7	KSS‐function	50.0	86.9	1.93	1.26	2.60	<0.001	870	3
Femoral flexion protocol 2	86.0	89.0	SF‐12	40.0	56.0	0.67	0.67	0.67	<0.001	400	1
Femoral flexion protocol 2	86.0	89.0	KOOS	45.0	87.0	1.42	0.62	2.21	0.011	400	1
Femoral sagittal angle 1	1.30	3.8‐	KSS‐knee	80.5	97.4	−6.09	−7.63	−4.54	<0.001	280	2
Posterior condylar offset	24.0	33.6	KSS‐overall	85.0	178	31.6	23.5	39.8	<0.001	2338	5

Abbreviations: FJS, forgotten joint score; KSS, knee society score; OKS, Oxford knee score; SF‐12; short‐form‐12‐health‐survey; TKA, total knee arthroplasty; WOMAC, Western Ontario and McMaster Universities Osteoarthritis Index.

^a^ *p*‐Value refers to the significance of the regression coefficient for association between the sagittal alignment parameter and the PROM.

#### Femoral sagittal angle (FSA)

FSA1 was reported in four studies (*n* = 494 TKRs), with PROMs assessed from one to 39 months. At 12 months, lower FSA1 was significantly associated with improved KSS‐Knee (RC = ‐6.20, *p* = 0.008, *n* = 160, FSA1 range: 1.53–3.80). When pooled across all time points, lower FSA1 was also associated with improved KSS‐knee (RC = –5.32, *p* < 0.001, *n* = 280, FSA1 range: 1.30–3.80) and EQ‐5D (RC = –0.21, *p* = 0.042, *n* = 180, FSA1 range: 3.20–3.80) (Figure 2c). Furthermore, time‐adjusted aggregated analysis showed lower FSA1 remained significantly associated with improved KSS‐knee (RC = –6.09, *p* < 0.001, *n* = 280, FSA1 range: 1.30–3.80) (Figure 3e).

#### Posterior condylar offset (PCO)

PCO was reported in 10 studies (*n* = 3383 TKRs), with PROMs assessed from 1 to 156 months. At 12 months, higher PCO was associated with improved KSS‐overall (RC = 42.945, *p* = 0.002, *n* = 613 TKRs, PCO range: 30.40–33.60 mm).

When pooled across all timepoints, higher PCO was associated with higher KSS‐knee (RC = 0.67, *p* = 0.019, *n* = 175, PCO range: 24.5–33.6 mm) but worse WOMAC‐total scores (RC = 8.64, *p* < 0.001, *n* = 2895, PCO range: 24.0–30.5 mm) (Figure 2d,e). Further, larger PCO ratio (PCOR; PCO: overall femoral size) was associated with worse WOMAC‐Total across all timepoints (RC = 62.2, *p* < 0.001, *n* = 617, PCOR range: 0.46–1.49).

Time‐adjusted aggregated analysis showed that higher PCO was associated with improved KSS‐overall (RC = 31.64, *p* < 0.001, *n* = 2338, PCO range: 24.0–33.6 mm) (Figure 3f).

#### Anterior condylar offset (ACO)

ACO was reported in four studies, with PROMs assessed from 1 to 30 months. When pooled across all timepoints, higher ACO was associated with higher WOMAC‐Total (RC = –2.43, *p* = 0.039, *n* = 1010; ACO range: 4.19–29.50 mm) (Figure 2f). However, no statistically significant relationship with any PROM was observed at individual timepoints or in the time‐adjusted aggregated analyses.

### Distal femoral sagittal anteverted angle (DFSAA) and flexion‐extension angle (FEA)

Only one study reported DFSAA or FEA and did not provide any statistical estimate of associate (e.g., regression coefficients), hence, it was not possible to assess any association with PROMs.

#### Sensitivity analyses

Sensitivity analyses, excluding studies at high risk of bias, demonstrated that the main associations largely persisted, with higher FF2, PCO, ACO and lower PTS remaining linked to better PROMs (Appendix S7).

## DISCUSSION

This systematic review and correlation analysis included 52 studies and found that most assessed only one or two sagittal TKR parameters, most commonly PTS and PCO. Moreover, the measurement protocols for several parameters were inconsistent, particularly for FF and FSA. Based on the available data, we found that sagittal alignment is associated with patient outcomes, specifically higher PTS, higher FF, lower FSA and higher PCO were all associated with PROMs representing better patient states.

PTS was the most frequently reported sagittal parameter and is consistently identified in the literature as an important determinant of knee mechanics and outcomes [9]. Our findings that increased PTS was associated with improved PROMs are consistent with prior work. Guo et al. [18] demonstrated in a simulation study that higher PTS improved knee kinematics and stability by facilitating posterior femoral translation and rollback. Similarly, colleagues [57] showed in cadaveric models that larger PTS increased posterior translation, enlarged contact area, and reduced pressures, improving stability and range of motion. Ogawa et al. [38] later translated these findings to patient outcomes, showing that increased PTS was associated with statistically significantly better symptoms and higher satisfaction scores. Current surgical practice commonly aims for 0°–7° PTS for most prostheses [16], yet our findings suggest that higher values may yield better outcomes. Similarly, we found that femoral component sagittal orientation, as measured by FF and FSA, influences PROMs. Previous studies have shown that positioning the femoral component in mild flexion (0°–3°) is associated with better knee ROM and KSS scores at 6 and 12 months [31, 61], whereas deviations into excessive flexion or extension impair function [36, 37, 56]. Our correlation‐analysis supports these findings: higher FF and lower FSA values (reflecting greater flexion) within observed ranges were associated with improved PROMs at 12 months and in pooled, time‐adjusted analyses. Although some statistically significant associations were observed within narrow alignment ranges, the clinical relevance of the small angular differences (e.g., within 1°–2°) requires cautious interpretation, as it is unclear if they translate to meaningful clinical improvements. Nevertheless, these findings reinforce that careful attention to PTS, FF and FSA, is important for optimising PROMs after TKR and highlight areas for further research.

PCO, representing the posterior thickness of the femoral condyles, is crucial for maintaining knee alignment, range of motion, and mid‐flexion stability, and that both reductions and excessive increases can impair function [32, 59, 60]. Our correlation analysis similarly found that higher absolute PCO within 30.4–33.6 mm was associated with better KSS‐overall at 12 months, and higher absolute PCO within 24.5–33.6 mm improved KSS‐knee and KSS‐overall in pooled and time‐adjusted analyses. Where limited literature exists, we show that higher PCOR, which adjusts PCO for femoral size, was associated with worse WOMAC scores across all timepoints, highlighting the mixed relationship between absolute and relative measures and PROMs. Because PCO depends on femoral and implant size, PCOR is likely an important sagittal parameter to consider as well as PCO. Regarding ACO, Scott et al. [44] showed that excessive increases elevate patellofemoral contact pressures and risk anterior knee pain, whereas moderate changes have minimal impact on PROMs. In our pooled analysis, higher ACO was associated with a small increase in WOMAC scores, but no consistent associations were seen at individual time points or in time‐adjusted analyses, likely reflecting limited reporting. Overall, these findings overall reinforce that careful attention to femoral condylar geometry, including PCO, PCOR and ACO, is required to optimise PROMs after TKR.

Our study has several key strengths. It adopts a comprehensive and systematic approach, including 52 studies with over 11,000 TKRs and 9400 patients, providing a large and diverse dataset. To our knowledge, this is the first correlation‐analysis which quantitatively demonstrates the association between post‐operative sagittal alignment and PROMs, providing a novel overview and identifying priorities for standardisation in future research. By simultaneously evaluating multiple sagittal alignment parameters (PTS, FF, FSA, PCO, PCOR and ACO) and their associations with a wide range of validated PROMs (KSS, OKS, WOMAC, FJS and others), our analysis provides a clinically relevant, patient‐centred understanding of how sagittal component positioning influences outcomes. The use of pooled and time‐adjusted aggregated analyses accounts for variations across follow‐up timepoints and allows assessment of temporal effects. Further, although a high proportion of included studies were observational cohorts with serious or critical risk of bias, we conducted a sensitivity analysis excluding these studies. This showed that the main associations persisted, strengthening confidence in our findings. Additionally, our study highlights methodological variability in current research, including differences in measurement protocols, omission of key parameters such as PTS, and predominant use of radiographs rather than CT, offering guidance for improving future study design. Collectively, these findings emphasise that optimising sagittal alignment should be a key focus for improving patient‐reported outcomes after TKR, particularly with increasing precision enabled by modern surgical technologies.

However, there are limitations to our review. First, the highly fragmented nature of reporting, with no study reporting all six sagittal parameters, prevented multivariate regression analyses and evaluation of inter‐relationships with PROMs. Second, definitions were inconsistent, particularly for FF and FSA which were measured against different reference points (e.g., anterior flange, bottom of femoral component, or perpendicular line) [14, 36, 55]. Although we split analyses by protocol to address this, it reduced the number of studies pooled and some associations (e.g., FF Protocol 3, FSA Protocols 2 and 3) were no longer statistically significant. Third, measurement methods varied across studies, with most relying on radiographs, which have been shown to be less accurate and reproducible than CT for both femoral and tibial sagittal alignment measurements, whereas CT with 3D reconstruction provides more precise assessment [47]. This measurement imprecision contributes to heterogeneity and may obscure or distort true associations. Fourth, although knee function, load distribution, kinematics and ligament tension depend on the interplay of all three planes [1, 5, 54], none of the studies in our review reported coronal, sagittal and axial alignment parameters together, limiting assessment of multiplanar interactions and their combined impact on PROMs. Further, recent evidence demonstrates the distraction forces required to achieve ligament balance in TKA, providing a mechanistic link between alignment targets and soft‐tissue status [3]. As sagittal component positioning directly influences ligament tension and soft‐tissue balance, the lack of studies reporting both alignment parameters and soft‐tissue measures limits evaluation of their combined effect on patient‐reported outcomes. Fifth, only 38% of studies reported the alignment method used, making it unclear whether observed associations reflect the parameters themselves or the alignment method, reducing clinical applicability. Sixth, it is important to note that these study‐level regression analyses are susceptible to ecological fallacy and residual confounding. Consequently, the reported findings indicate associations at the study population level rather than confirming individual patient‐level relationships or prescriptive alignment targets.

## CONCLUSIONS

To our knowledge, this is the first correlation analysis demonstrating that post‐operative sagittal alignment is associated with patient outcomes, and we encourage surgeons to consider sagittal alignment alongside coronal alignment when planning and performing TKRs. Our review suggests that higher PTS, higher FF, lower FSA and higher PCO within observed ranges are associated with improved PROMs, although results vary across timepoints and are study‐level. These inconsistencies likely reflect heterogeneity in definitions, measurement protocols, reporting and reliance on radiographs. Future research should address these limitations identified in our review by developing standardised definitions, protocols and a uniform reporting framework for alignment parameters in all three planes and utilising CT for more accurate assessment. These steps will enable the generation of comparable datasets, facilitate robust meta‐analyses to identify the parameters most strongly influencing PROMs in each plane and reduce research waste. Such work is essential to advance evidence‐based alignment methods in TKR, ultimately guiding clinical decision‐making and improving patient outcomes.

## AUTHOR CONTRIBUTIONS

*Guarantor of integrity of the entire study*: Prakrit R. Kumar, Daya K. Sumra, Aurora Segre Carnell, Ranya V. Kumar, Amr Selim, Muhamed M. Farhan‐Alanie, Helen Parsons and Andrew Metcalfe. *Study concepts and design*: Prakrit R. Kumar, Daya K. Sumra, Aurora Segre Carnell, Ranya V. Kumar, Amr Selim, Muhamed M. Farhan‐Alanie, Helen Parsons and Andrew Metcalfe. *Literature research*: Prakrit R. Kumar, Daya K. Sumra, Aurora Segre Carnell, Ranya V. Kumar, Umar Said, Amr Selim and Muhamed M. Farhan‐Alanie. *Experimental studies/data analysis*: Prakrit R. Kumar, Daya K. Sumra, Aurora Segre Carnell, Ranya V. Kumar, Amr Selim and Muhamed M. Farhan‐Alanie. *Statistical analysis*: Prakrit R. Kumar, Daya K. Sumra, Aurora Segre Carnell, Ranya V. Kumar, Amr Selim and Muhamed M. Farhan‐Alanie. *Manuscript preparation*: Prakrit R. Kumar, Daya K. Sumra, Aurora Segre Carnell, Ranya V. Kumar, Umar Said, Amr Selim, Muhamed M. Farhan‐Alanie, Helen Parsons and Andrew Metcalfe. *Manuscript editing*: Prakrit R. Kumar, Daya K. Sumra, Aurora Segre Carnell, Ranya V. Kumar, Umar Said, Amr Selim, Muhamed M. Farhan‐Alanie, Helen Parsons and Andrew Metcalfe. *Manuscript revisions*: Prakrit R. Kumar, Daya K. Sumra, Aurora Segre Carnell, Ranya V. Kumar, Umar Said, Amr Selim, Muhamed M. Farhan‐Alanie, Helen Parsons and Andrew Metcalfe.

## CONFLICT OF INTEREST STATEMENT

Andrew Metcalfe is Chief Investigator and Helen Parsons is the trial statistician for the RACER trial. They are an investigators on three National Institute for Health and Care Research (NIHR)‐funded trials (Andrew Metcalfe is Chief Investigator for START:REACTS and RACER‐Knee and co‐investigator for RACER‐Hip, Helen Parsons is co‐applicant and trial statistician on each), for which Stryker, a medical device company, fund some treatment costs and some imaging costs. For all these studies, the full independence of the study team is protected by legal agreements and they have no bearing on the presented study. The remaining authors declare no conflicts of interest.

## ETHICS STATEMENT

The authors have nothing to report.

## Supporting information

Supporting File 1

Supporting File 2

Supporting File 3

Supporting File 4

Supporting File 5

Supporting File 6

Supporting File 7

## Data Availability

The data supporting the findings of this study are available in the figures, tables and supplementary material of this article.
