# Supplementary material for: Effect of sagittal alignment on patient outcomes following total knee replacement: A systematic review and correlation analysis
Source: J Exp Orthop. 2026 May 4;13(2):e70731. doi: 10.1002/jeo2.70731 (PMC13137439; doi:10.1002/jeo2.70731)
Supplement: Supplementary file 3 — Supporting File 3 [file JEO2-13-e70731-s003.docx]

| **Appendix 3:**  **Seach terms deployed conduct literature searches of Scopus, Web of Science, Pubmed, Embase, and Cochrane Central Register of Controlled Trials** | | | | |
| --- | --- | --- | --- | --- |
| **Database** | **Search String** | | | **Hits (n studies)** |
| **Scopus** | ( ( TITLE-ABS-KEY ( "total knee replacement" OR "total knee arthroplasty" OR “tkr” OR “tka” )    AND TITLE-ABS-KEY ( "Posterior Tibia* Slope" OR "Tibia* Slope"  OR “Tibia sagittal angle” OR  “PTS" OR “TS” OR “TSA” OR “Femoral Flexion” OR “FF” OR “Femoral Sagittal Angle” OR “Flexion extension angle” OR “FSA” OR “FE angle” OR “Anterior condylar offset” OR “ACO” OR “Posterior condylar offset” OR “PCO”)    AND TITLE-ABS-KEY ( “PROM” OR “patient outcome” OR "treatment outcome" OR "western ontario and mcmaster & universities osteoarthritis index" OR “WOMAC” OR "oxford knee score" OR “OKS” OR "hospital for special surgery score" OR “hss” OR "knee society score" OR “kss” OR "forgotten joint score" OR “fjs” OR “euroqol 5” OR "euroqol-5" OR “Short form 12” OR “Short form-12” OR “SF-12” OR “SF 12” OR “Short form 36” OR “Short form-36” OR “SF-36” OR “SF 36” OR “Knee Injury and Osteoarthritis Outcome Score” OR “KOOS”) ) ) | | | 263 |
| **Web of Science** | ( ( TITLE-ABS-KEY ( "total knee replacement" OR "total knee arthroplasty" OR “tkr” OR “tka” )    AND TITLE-ABS-KEY ( "Posterior Tibia* Slope" OR "Tibia* Slope"  OR “Tibia sagittal angle” OR  “PTS" OR “TS” OR “TSA” OR “Femoral Flexion” OR “FF” OR “Femoral Sagittal Angle” OR “Flexion extension angle” OR “Flexion/extension angle” OR “FSA” OR “FE angle” OR “F/E angle” OR “Anterior condylar offset” OR “ACO” OR “Posterior condylar offset” OR “PCO”)    AND TITLE-ABS-KEY ( “PROM” OR “patient outcome” OR "treatment outcome" OR "western ontario and mcmaster & universities osteoarthritis index" OR “WOMAC” OR "oxford knee score" OR “OKS” OR "hospital for special surgery score" OR “hss” OR "knee society score" OR “kss” OR "forgotten joint score" OR “fjs” OR “euroqol 5” OR "euroqol-5" OR “Short form 12” OR “Short form-12” OR “SF-12” OR “SF 12” OR “Short form 36” OR “Short form-36” OR “SF-36” OR “SF 36” OR “Knee Injury and Osteoarthritis Outcome Score” OR “KOOS”) ) ) | | | 159 |
| **Pubmed (P)/**  **Embase (E)/**  **Cochrane Central Register of Controlled Trials (C)** | 1. total knee replacement.mp. or exp Arthroplasty, Replacement, Knee/ 2. total knee arthroplasty.mp. 3. tka.mp. 4. tkr.mp. 5. 1 or 2 or 3 or 4 6. Posterior Tibia* Slope.mp. 7. Tibia* Slope.mp. 8. Tibia sagittal angle.mp. 9. PTS.mp. 10. TS.mp. 11. TSA.mp. 12. Femoral Flexion.mp. 13. FF.mp. 14. Femoral Sagittal Angle.mp. 15. Flexion extension angle.mp. 16. Flexion/extension angle.mp. 17. FSA.mp. 18. FE angle.mp. 19. F/E angle.mp. 20. Anterior condylar offset.mp. | 1. ACO.mp. 2. Posterior condylar offset.mp. 3. PCO.mp. 4. 6 or 7 or 8 or 9 or 10 or 11 or 12 or 13 or 14 or 15 or 16 or 17 or 18 or 19 or 20 or 21 or 22 or 23 5. 5 and 24 6. Patient outcome assessment.mp. 7. PROM.mp. 8. Patient outcome.mp. 9. Treatment outcome.mp. 10. WOMAC.mp. 11. Western Ontario and McMaster's Universities Osteoarthritis Index.mp. [mp=ti, ab, hw, tn, ot, dm, mf, dv, kf, fx, dq, bt, nm, ox, px, rx, ui, sy, ux, mx] 12. oxford knee score.mp. 13. oks.mp. 14. hospital for special surgery score.mp. 15. hss.mp. 16. knee society score.mp. 17. kss.mp. 18. forgotten joint score.mp. | 1. fjs.mp. 2. Euroqol 5.mp. 3. Euroqol-5.mp. 4. Short-form 12.mp. 5. Short form 12.mp. 6. SF-12.mp. 7. SF 12.mp. 8. Short-form 36.mp. 9. Short form 36.mp. 10. SF-36.mp. 11. SF 36.mp. 12. Knee Injury and Osteoarthritis Outcome Score.mp. 13. KOOS 14. 26 or 27 or 28 or 29 or 30 or 31 or 32 or 33 or 34 or 35 or 36 or 37 or 38 or 39 or 40 or 41 or 42 or 43 or 44 or 45 or 46 or 47 or 48 or 49 or 50 or 51 15. 24 and 52 16. Limit 53 to English language | P: 234  E: 281  C: 257 |
