# Supplementary material for: Effect of sagittal alignment on patient outcomes following total knee replacement: A systematic review and correlation analysis
Source: J Exp Orthop. 2026 May 4;13(2):e70731. doi: 10.1002/jeo2.70731 (PMC13137439; doi:10.1002/jeo2.70731)
Supplement: Supplementary file 4 — Supporting File 4 [file JEO2-13-e70731-s005.pptx]

## Slide 1
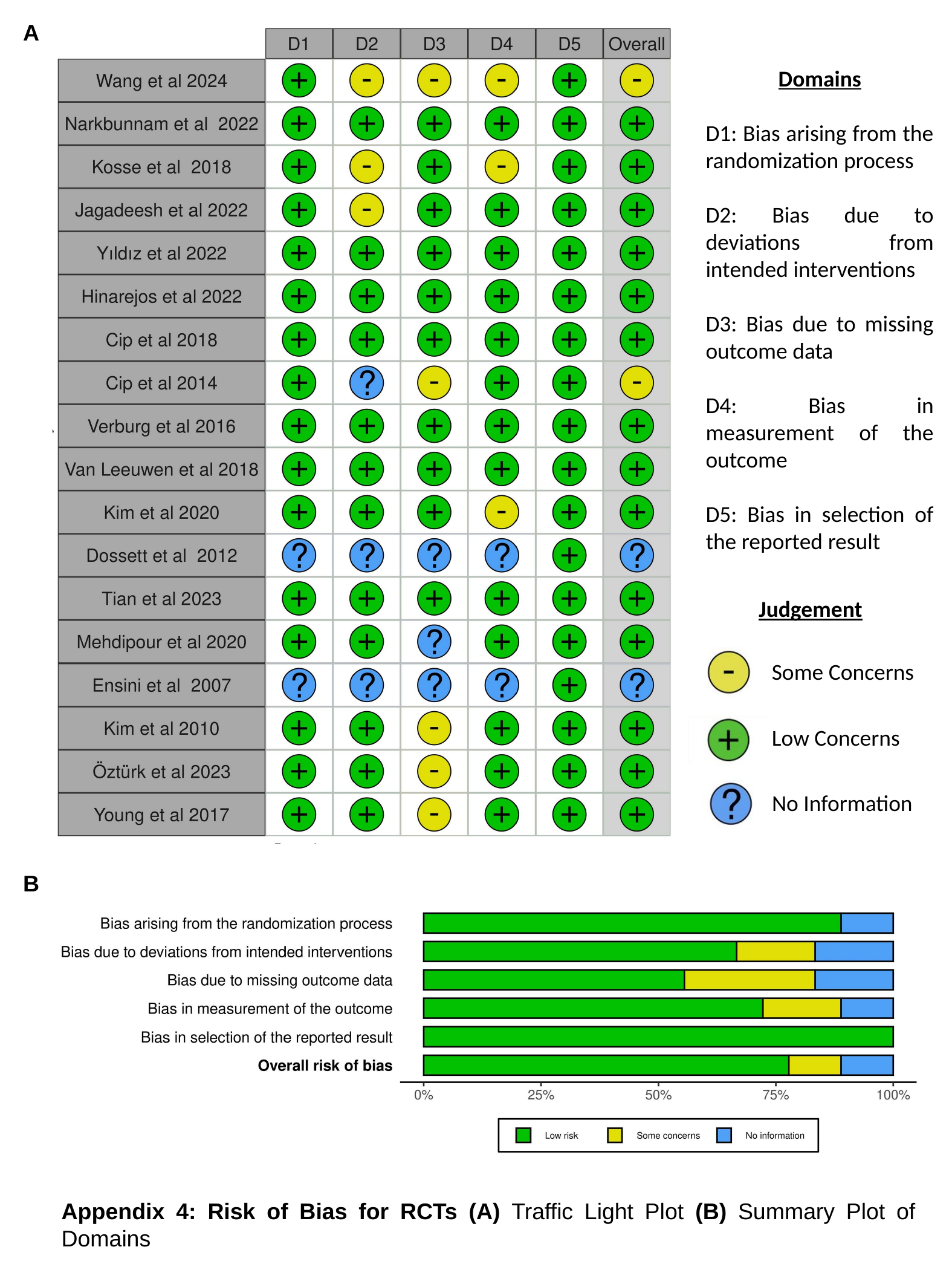

A
Domains
D1: Bias arising from the randomization process
D2: Bias due to deviations from intended interventions
D3: Bias due to missing outcome data
D4: Bias in measurement of the outcome
D5: Bias in selection of the reported result
Judgement
Some Concerns
Low Concerns
No Information
B
Appendix 4: Risk of Bias for RCTs (A) Traffic Light Plot (B) Summary Plot of Domains
