# Supplementary material for: Effect of sagittal alignment on patient outcomes following total knee replacement: A systematic review and correlation analysis
Source: J Exp Orthop. 2026 May 4;13(2):e70731. doi: 10.1002/jeo2.70731 (PMC13137439; doi:10.1002/jeo2.70731)
Supplement: Supplementary file 5 — Supporting File 5 [file JEO2-13-e70731-s007.pptx]

## Slide 1
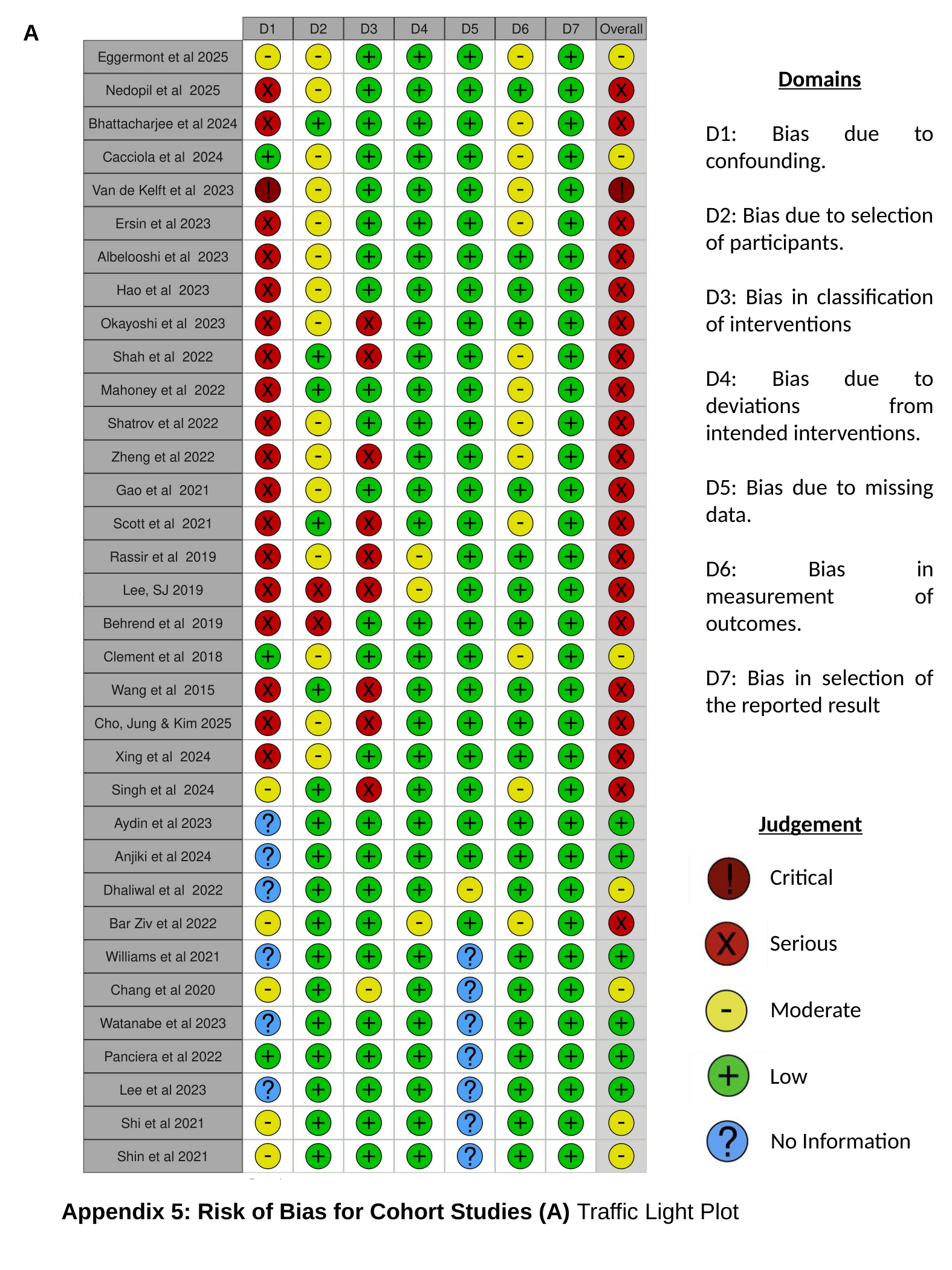

A
Domains
D1: Bias due to confounding.
D2: Bias due to selection of participants.
D3: Bias in classification of interventions
D4: Bias due to deviations from intended interventions.
D5: Bias due to missing data.
D6: Bias in measurement of outcomes.
D7: Bias in selection of the reported result
Judgement
Critical
Serious
Moderate
Low
No Information
Appendix 5: Risk of Bias for Cohort Studies (A) Traffic Light Plot

## Slide 2
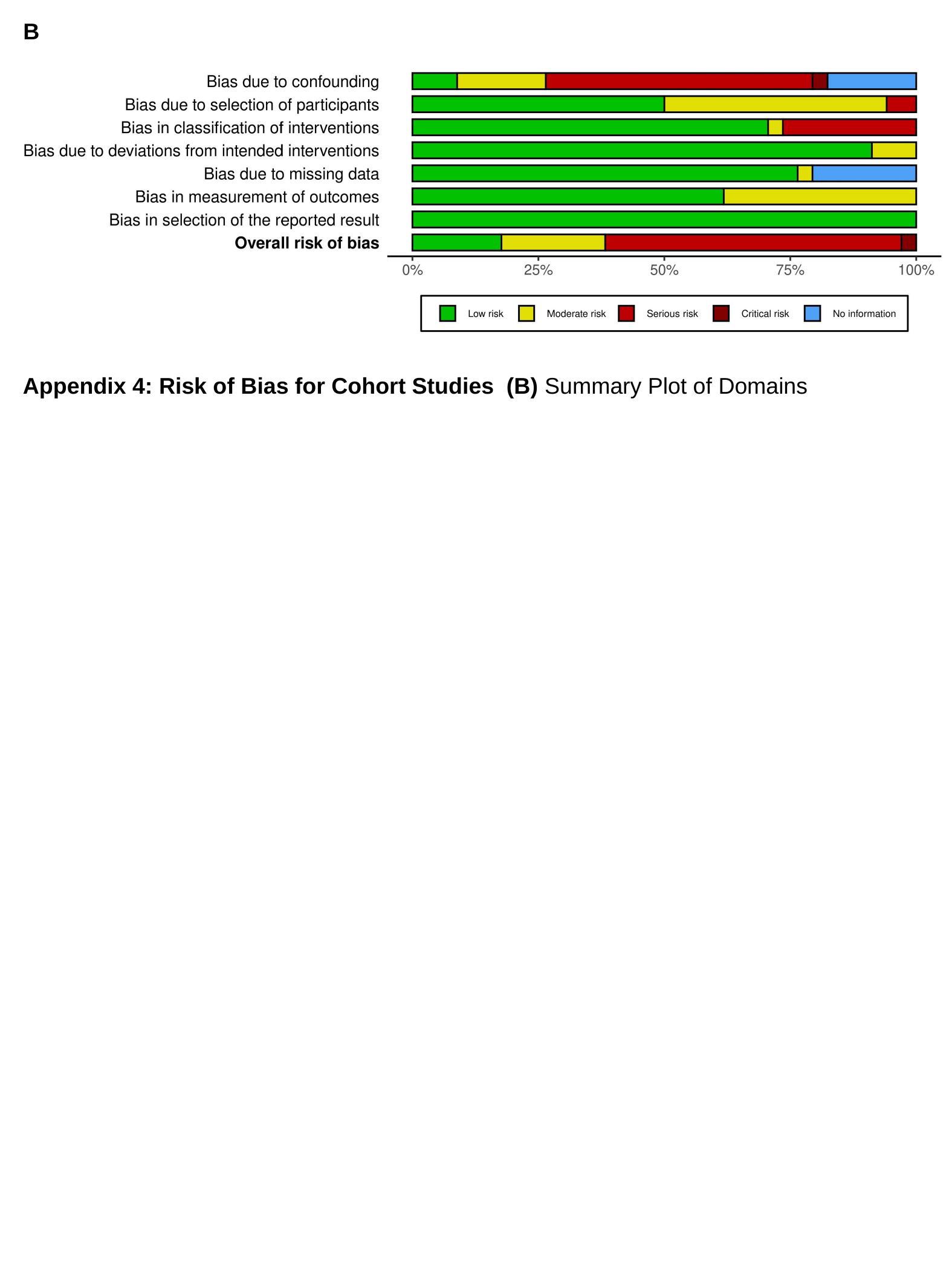

B
Appendix 4: Risk of Bias for Cohort Studies (B) Summary Plot of Domains
