# Supplementary material for: Effect of sagittal alignment on patient outcomes following total knee replacement: A systematic review and correlation analysis
Source: J Exp Orthop. 2026 May 4;13(2):e70731. doi: 10.1002/jeo2.70731 (PMC13137439; doi:10.1002/jeo2.70731)
Supplement: Supplementary file 6 — Supporting File 6 [file JEO2-13-e70731-s002.docx]

| **Appendix 6: Sagittal Alignment Parameters Definitions** | | |
| --- | --- | --- |
| **Sagittal Parameter** | **Description** | **Number of Studies** |
| Posterior Tibia Slope | Angle between the longitudinal axis of the tibia and the posterior inclination of the tibial plateau | 39 |
|  |  |  |
| Distal Femoral Sagittal Anteverted Angle | Angle formed by projecting the femoral mechanical axis and distal femoral anatomical axis onto the femur’s sagittal plane. | 1 |
|  |  |  |
| Femoral Flexion Angle Protocol 1 | Angle between the anatomical axis of the distal femur and the anterior flange of the femoral prosthesis. | 9 |
|  |  |  |
| Femoral Flexion Angle Protocol 2 | Angle between the femoral anatomical axis and the bottom of the femoral component | 4 |
|  |  |  |
| Femoral Flexion Angle Protocol 3 | Angle formed between the anatomical axis of the femur and a perpendicular line to the femoral prosthesis. | 2 |
|  |  |  |
| Femoral Sagittal Angle Protocol 1 | Angle between the distal femoral prosthesis line and the femoral mechanical axis | 4 |
|  |  |  |
| Femoral Sagittal Angle Protocol 2 | Posterior angle between the anterior cortical axis (line linking two points on anterior cortex 5 and 15cm proximal to joint line) and the slope of the distal femoral cut. | 1 |
|  |  |  |
| Femoral Sagittal Angle Protocol 3 | Angle between a line from the femoral head centre to the deepest notch point and a line across the posterior flange of the femoral component. | 1 |
|  |  |  |
| Flexion Extension Angle | Anterior angle between the femoral anatomical axis and the distal femoral cutting line. | 1 |
|  |  |  |
| Posterior Condylar Offset | Distance between the posterior femoral cortex and the most posterior aspect of the femoral condyles. | 10 |
|  |  |  |
| Posterior Condylar Offset Ratio | Ratio of the posterior condylar offset relative to the overall femoral size. | 7 |
|  |  |  |
| Anterior Condylar Offset | Distance from the anterior cortex of the femur to the anterior aspect of the femoral component. | 4 |
