# Supplementary material for: Effect of sagittal alignment on patient outcomes following total knee replacement: A systematic review and correlation analysis
Source: J Exp Orthop. 2026 May 4;13(2):e70731. doi: 10.1002/jeo2.70731 (PMC13137439; doi:10.1002/jeo2.70731)
Supplement: Supplementary file 7 — Supporting File 7 [file JEO2-13-e70731-s006.docx]

**Appendix 7: Sensitivity Analysis**

Sensitivity analyses were performed by removing studies at high risk of bias studies. We excluded 4 RCTs with *“some concerns/no information”* and 21 cohorts with *“serious/critical”* bias, leaving 14 RCTs and 13 cohorts at *“low/moderate”* risk of bias.

**At individual timepoints:**

- Higher PCO remained associated with improved KSS-Overall at 12 months (*RC = 43.95, p = 0.002, n = 613; PCO range: 30.40–33.60*).

**When pooled across all timepoints:**

- Lower PTS remained associated with better EuroQol-5D (*RC = –10.33, p = 0.042, n = 180; PTS range: 3.60–3.80*).
- Lower FSA1 remained associated with better EuroQol-5D (*RC = –3.44, p = 0.042, n = 180; FSA1 range: 3.20–3.80*).
- Higher PCO remained associated with worse WOMAC-Total (*RC = 8.66, p < 0.001, n = 2655; PCO range: 24.00–30.50*).
- Higher ACO remained associated with better WOMAC-Total (*RC = –3.78, p = 0.003, n = 770; ACO range: 4.19–29.50*).

**In time-adjusted analysis:**

- Higher FF2 (*range: 86.0–90.7*) remained associated with improved outcomes across multiple measures:
  - KSS-Overall (*RC = 0.83, p = 0.003, n = 510*)
  - KSS-Function (*RC = 1.58, p < 0.001, n = 510*)
  - SF-12 (*RC = 0.67, p < 0.001, n = 400*)
  - KOOS (*RC = 1.42, p = 0.011, n = 400*).
- Lower FSA1 remained associated with better KSS-Knee (*RC = –0.315, p = 0.040, n = 180; FSA1 range: 3.20–3.80*).
- Higher PCO remained associated with better KSS-Overall (*RC = 31.64, p < 0.001, n = 2338; PCO range: 24.00–33.60*).
